# Safety and feasibility of breast lesion localization using magnetic seeds (Magseed): a multi-centre, open-label cohort study

**DOI:** 10.1007/s10549-018-4709-y

**Published:** 2018-02-16

**Authors:** James R. Harvey, Yit Lim, John Murphy, Miles Howe, Julie Morris, Amit Goyal, Anthony J. Maxwell

**Affiliations:** 1 Manchester Academic Health Science Centre, Nightingale Centre, University Hospital of South Manchester, Wythenshawe Hospital, University of Manchester, Southmoor Road, Manchester, M23 9LT UK; 2 Royal Derby Hospital, Derby, Derbyshire DE22 3NE UK; 3 Division of Informatics, Imaging & Data Sciences, School of Health Sciences, Faculty of Biology, Medicine and Health, University of Manchester, Manchester, M13 9PT UK

**Keywords:** Mastectomy, Breast cancer, Lumpectomy, Seed localization, Magnetic

## Abstract

**Purpose:**

Wire localization has several disadvantages, notably wire migration and difficulty scheduling the procedure close to surgery. Radioactive seed localization overcomes these disadvantages, but implementation is limited due to radiation safety requirements. Magnetic seeds potentially offer the logistical benefits and transcutaneous detection equivalence of a radioactive seed, with easier implementation. This study was designed to evaluate the feasibility and safety of using magnetic seeds for breast lesion localization.

**Methods:**

A two-centre open-label cohort study to assess the feasibility and safety of magnetic seed (Magseed) localization of breast lesions. Magseeds were placed under radiological guidance into women having total mastectomy surgery. The primary outcome measure was seed migration distance. Secondary outcome measures included accuracy of placement, ease of transcutaneous detection, seed integrity and safety.

**Results:**

Twenty-nine Magseeds were placed into the breasts of 28 patients under ultrasound guidance. There was no migration of the seeds between placement and surgery. Twenty-seven seeds were placed directly in the target lesion with the other seeds being 2 and 3 mm away. All seeds were detectable transcutaneously in all breast sizes and at all depths. There were no complications or safety issues.

**Conclusions:**

Magnetic seeds are a feasible and safe method of breast lesion localization. They can be accurately placed, demonstrate no migration in this feasibility study and are detectable in all sizes and depths of breast tissue. Now that safety and feasibility have been demonstrated, further clinical studies are required to evaluate the seed’s effectiveness in wide local excision surgery.

## Introduction

Excision of impalpable breast lesions is usually directed by preoperative wire placement into or adjacent to the target lesion. Wire localization has several disadvantages, most notably, displacement of the wire, and difficulty in the surgeon discerning accurately the position of the tip of the wire intraoperatively [1]. The entry point of the wire may be some distance from the wire tip, making optimal incision placement a challenge and leading to extensive dissection to remove the target lesion. Additionally, wire placement occurs on the day of surgery which can create problems for radiology and surgery scheduling and lead to delays in the operating theatre. However, it remains the default method of localization due to the limitations of other methods of localization and given the long-term data supporting its effectiveness [2].

Iodine (^125^I) radioactive seed localization is used in some centres to overcome many of the disadvantages of wire localization. It can be performed prior to the day of surgery and the surgeon can accurately localize the device in theatre using a hand-held gamma probe, providing major logistical advantages [3]. However, the radiation safety precautions required to set up and support this service limits its widespread implementation [4]. Radioactive seed localization and radiooccult lesion localization (ROLL) are equally reliable to wire localization [2]. ROLL offers less logistical advantage compared to seeds, because it still requires patient injection of radioisotope into the tumour bed to occur within 24 h of surgery. Unless contrast is also given, ROLL does not offer the surgeon mammographic or ultrasound confirmation of the site of injection in relation to the lesion [5].

**Fig. 1:**
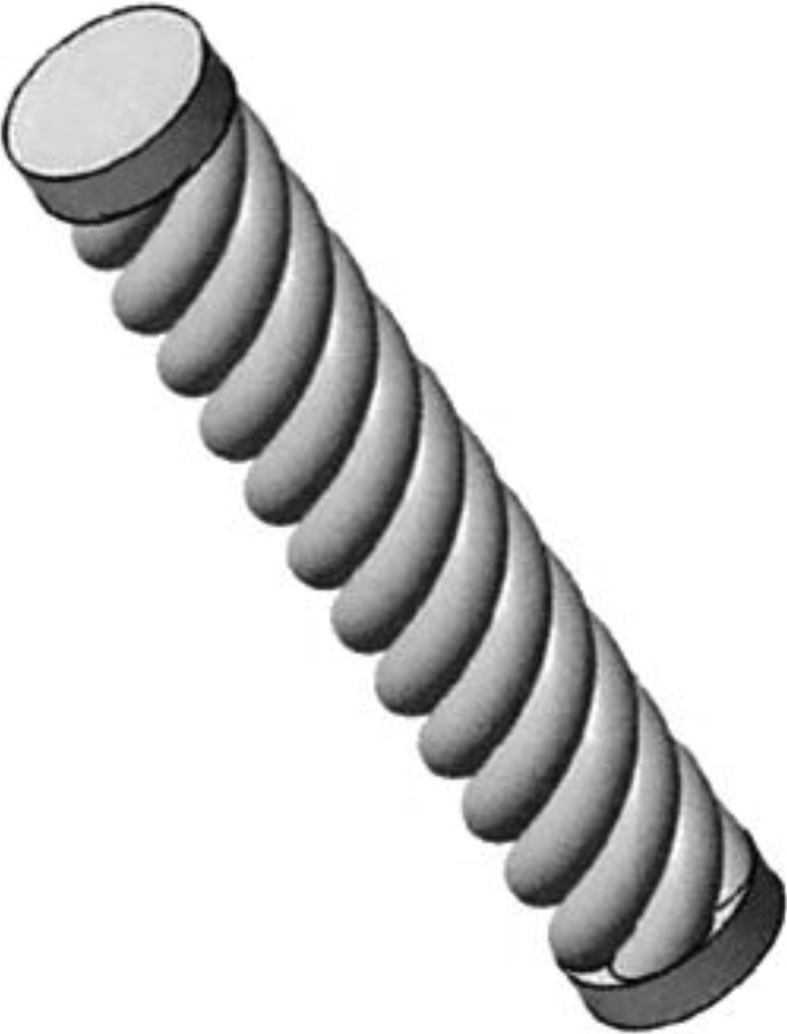
Magseed Structure—Magseed consists of a 5 × 1 mm paramagnetic steel seed which does not have the barbs of a traditional wire used for localization

Magseed is an alternative method of localizing breast lesions. It consists of a 5 × 1 mm paramagnetic steel and iron oxide seed (Fig. 1). The seed is cylindrical with no barbs and is readily visible on mammography and ultrasound. It is supplied in sterile packaging preloaded into an 18-gauge 20-cm-long steel needle. The seed is retained by a wax plug and there is a steel obturator which is advanced to deploy the seed. The seed is detectable using the Sentimag probe in the same way as the Sienna dye [6] used in sentinel lymph node biopsy. The probe generates an alternating magnetic field which transiently magnetizes the iron oxide particles within the Magseed. The magnetic signature of the Magseed is then detected by the Sentimag probe. The Sentimag unit displays a numerical count and produces an audio tone, which are related to the strength of the magnetic field and therefore the distance of the seed from the detector probe. Magseed offers the potential advantages of a radioactive seed without the onerous radiation governance requirements.

Although it is already in use in some centres in the USA, this is the first clinical study of Magseed lesion localization. The aim of the study was to assess the safety and feasibility of Magseed localization of breast lesions. The objectives included measurement of seed migration, ease of Magseed detection in a range of breast sizes and at various depths and assessment of any tissue reaction. The study was performed in women scheduled to undergo total mastectomy.

## Methods

### Patients

Patients were enrolled in this multi-centre, open-label cohort study between July 2016 and April 2017 at two University Hospitals. The inclusion criteria were female patients, age 18 or older, with a core biopsy-proven breast cancer (invasive carcinoma or ductal carcinoma-in situ) for which total mastectomy was planned. The exclusion criteria were a pacemaker or implanted device in the chest wall, nickel allergy, pregnancy/lactation, known coagulopathy or current anticoagulant medication, neoadjuvant chemotherapy and Sienna (iron oxide) injection in the previous 6 months. Patients were not offered seed placement until any necessary MRI scans had been performed.

The study was approved by the North West Research Ethics Committee (reference NW/16/0092) and was registered at www.clinicaltrials.gov (protocol record NCT02635737). Written informed consent was obtained from all participants.

### Procedure

Magseeds (Endomagnetics, Cambridge, UK) were inserted into the centre of the target lesions under local anesthetic and ultrasound guidance, a minimum of 2 days and a maximum of 30 days prior to surgery. The depth of the seed under the skin was measured with ultrasound using minimal breast compression with the patient supine and the ipsilateral arm abducted. Ipsilateral two-view mammography (mediolateral oblique and craniocaudal views) were performed immediately after the procedure to document the seed position. A Sentimag detector was used to check that the seed was detectable in the breast.

Repeat two-view mammography was performed on the day of surgery to confirm accurate positioning of the seed and to measure any migration. In the operating theatre, the surgeon localised the seed using the Sentimag detector and recorded the time taken to detect the seed together with the maximum recorded detector count. Total mastectomy surgery was performed followed by an x-ray of the mastectomy specimen to confirm seed removal. Histopathological examination of the Magseed site in the breast was performed for a sample of cases (the UK Medicines and Healthcare products Regulatory Agency stipulated that a minimum of ten resected specimens should be assessed for any tissue reactions to the seed).

### Outcomes

The primary outcome measure was an evaluation of the distribution of seed migration, to estimate the risk of markers migrating a clinically significant distance (10 mm or greater).

Secondary outcomes were accuracy of initial placement, relationship between depth of seed placement and ease of transcutaneous detection, seed integrity, safety and tolerability, total mastectomy weight and relationship between clinical characteristics and movement of the seed. To establish the detectability of the seed in all breast sizes, we set out to place seeds in a minimum of three each of small (< 250 g), and medium (250–500 g)-sized breasts and in five large (> 500 g) breasts. Assessment of complications was done from the time of seed placement until the post-operative visit to the outpatient clinic.

### Statistical analysis

Simple descriptive summary statistics of the main parameters were derived. Percentages for categorical variables, means, medians and range values for quantitative factors were calculated as appropriate. The relationship between breast size and detectability of the seed by the Sentimag detector was assessed using simple correlational analysis using Spearman correlation (rho). The statistical software package, SPSS version 22, was used for the statistical analysis.

## Results

Twenty-nine patients were recruited into the study; their baseline characteristics are shown in Table 1. Twenty-nine Magseed devices were placed into the breasts of 28 patients, 24 under ultrasound guidance and five under stereotactic guidance. One patient had bilateral seed placement. One patient had seed placement, but on the day of surgery failed to have her check mammograms to ascertain clip position and migration and did not have any intraoperative measurements taken, due to failure to identify the patient as a trial patient on the day of surgery. All Magseed devices were retrieved, as confirmed by specimen radiography, as were the target lesions.

**Table 1:** Baseline characteristics and pathological characteristics

Age (years); mean (range)	54 (37–75)
BMI (kg/m^2^); mean (range)	28.3 (20.3–42.2)
Lateralisation	
Right	11 (39%)
Left	16 (57%)
Bilateral	1 (4%)
Tumour stage	
Risk reducing	1 (3%)
Tis	3 (10%)
T1	12 (41%)
T2	9 (31%)
T3	4 (14%)
Invasive carcinomas	
Grade 1	3 (12%)
Grade 2	13 (52%)
Grade 3	9 (36%)
DCIS present	7
Invasive ductal carcinoma	19 (76%)
Invasive lobular carcinoma	5 (20%)
Mixed ductal/lobular carcinoma	1 (4%)
ER status	
Positive	24 (96%)
Negative	1 (4%)
Her 2 status	
Normal	23 (92%)
Over expression	2 (8%)

### Migration

No Magseed displacement between the two mammograms was observed in any of the participants, and thus 100% of the Magseeds showed migration within the acceptable target distance of less than 10 mm, 95% confidence interval (88–100%).

### Accuracy of placement

27/29 Magseeds were placed directly in the target lesion, 93%, (confidence interval 78–98%) the other two seeds were 2 and 3 mm from the target lesion, respectively. Magseeds were placed a median of 5 days before surgery (range 1–15 days).

Post-insertion ultrasound showed a median Magseed depth of 16 mm (range 3.5–30 mm).

All seeds were removed intact, as shown on specimen X-ray and on histology from 18 breasts.

### Safety and tolerability

There were no complications or adverse events recorded related to either the seed placement or to the surgery.

### Ease of detection

The Magseed was detectable with the Sentimag detector in all sizes of breasts. Mean breast weight was 833 g (range 126–2600 g), including four small (< 250 g), four medium (250–500 g), and 21 large (> 500 g) breasts). The Magseed was detectable at all depths as measured on ultrasound from 3.5 to 30 mm.

### Relationship between depth of seed placement and ease of transcutaneous detection

**Fig. 2:**
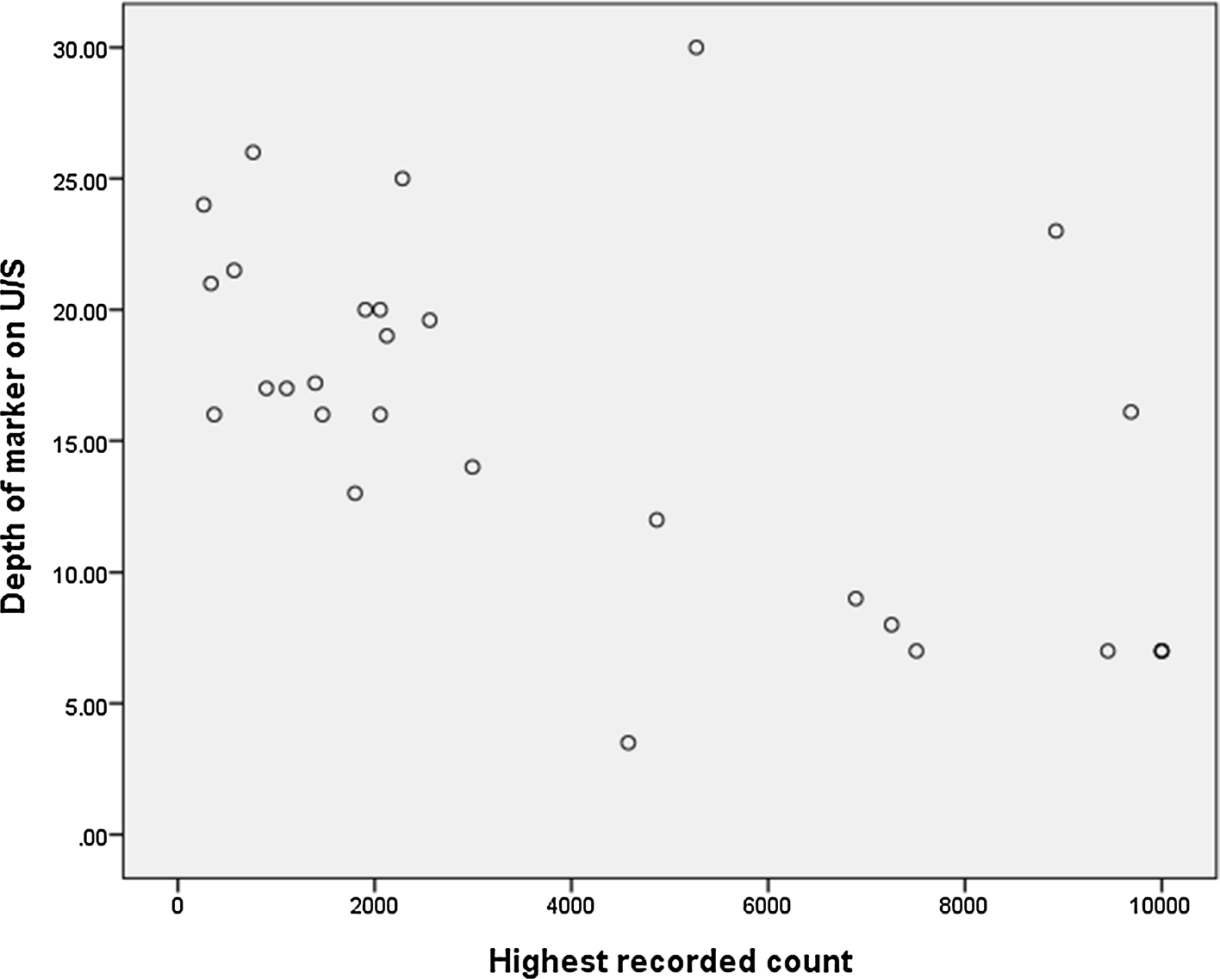
Spearman correlation between Depth of Magseed marker on Ultrasound and the Highest recorded count on the Sentimag detector. The deeper the seed is placed in the breast the lower the reading recorded on the Sentimag device and the longer it took to detect the device

The time taken to detect the seed was related its depth in the breast (rho = 0.38; *p* = 0.043), more superficial seeds being more quickly found with the Sentimag device. The more superficial the seed, the higher the recorded count on the device (Depth vs ‘highest recorded count’ rho = − 0.57; *p* = 0.001*) (Fig. 2). The ease of detection of the Magseed was also related to the size of the breast, with a correlation between breast weight and time to detect marker (rho = 0.29; *p* = 0.13) and between breast weight and the highest recorded count on the Sentimag probe (rho = − 0.54; *p* = 0.002*), with smaller breasts being faster to find the seed and with a higher initial count.

### Pathology reaction

Eighteen breasts had a pathological assessment to identify any tissue reaction to the Magseed. Nine seeds had no tissue reaction and nine showed evidence of mild inflammatory and foreign body reaction with fibrosis.

## Discussion

This feasibility study aimed to demonstrate that the Magseed device could be used for localization of breast cancers. The Sentimag probe that is used to detect Magseed generates an alternating magnetic field which transiently magnetises the iron particles within the Magseed. The magnetic signature of the Magseed device is then detected by the Sentimag probe. Due to the small size of the seed and hence containing only a small amount of paramagnetic material, there was uncertainty about whether it would be detectable by the probe at all depths in the breast and whether its use would be restricted to more superficial lesions or smaller breasts. Due to the uncertainties about its performance in clinical practice, the study was designed for women having mastectomies, so that the device performance could be assessed in a safe manner. If the device showed efficacy in total mastectomy patients, then Magseed could be evaluated in subsequent studies for its intended purpose of lumpectomy surgery. In clinical practice, 100% of the seeds used in this study were detectable using the Sentimag probe intraoperatively, in a wide range of breast sizes and at all depths. An alternative novel method of localization is the Savi Scout surgical guidance system which uses electromagnetic wave reflectors, but challenges were encountered in transcutaneous detection of this device in 2.5% of patients in a recent study [7]. Further study will be needed to ensure that the Magseed device is detectable at all depths, especially for posterior lesions in very large breasts.

Mamaloc is a similar technology using a magnetic seed to localize breast lesions which has shown similar feasibility outcomes in terms of migration and detectability in fifteen patients when co-localizing breast lesions with radioactive seeds [8]. The mechanism of action is similar between Mamaloc and Magseed; however, the Magseed’s composition has been designed to maximize the magnetic signal for the given size of seed. This enables the Magseed to be detectable in all breast sizes. Magseed is commercially available, has the regulatory approval in Europe for medical device safety (CE mark) and has been used for over 3000 localization procedures. It is therefore known to be a viable localization technique in all sizes of breast and this study forms the basis of its clinical safety profile for lumpectomy surgery. It is unclear whether Mamaloc will become available for widespread clinical use.

100% of the seeds were placed accurately within the target area. This compares favourably with 95% in radioactive seed localization and 89% in wire-guided localization [3]. However, further investigation of the seed’s performance in smaller screen-detected lesions is clearly mandated. Clinically, the strength of signal was related to the depth of the seed and in the larger breast, compression with the probe helped to locate the device. The surgeon is aware preoperatively of the location of the Magseed device from mammography and this aids rapid detection of the seed. The Sentimag probe gives a directional signal of the site of the marker seed which would enable lumpectomy removal of the tissue surrounding the seed.

The Magseed is similar in composition to a conventional radiological biopsy marker, and as expected there were minor foreign body reactions associated with the seeds on histopathology.

In Europe, the device was not CE marked for use until its clinical safety had been proven. European CE marking of the device was granted as a result of the safety data gathered during this feasibility study. However, in the US, FDA approval for the device was granted in 2016 and the device has been used for over 3000 breast localization procedures. Initial feedback from this (Endomagnetics) is positive and the device works as intended. Several large centres have adopted the technology as their primary means of localization, and we await publication of their results and experience.

The radiologists who inserted the seeds found them easy to deploy, similar to the insertion of a conventional biopsy marker. Apart from initial familiarization with the seed insertion system and the Sentimag device they did not require any specific training in either centre in order to place the seeds accurately.

One limitation of the device is its cost in comparison with wire and radioactive seed localization. However, we would envisage that the device will be logistically easier for radiological placement and would lead to a reduction in operating theatre delays, which could provide financial savings. The placement of this device in advance of surgery will also impact the patient journey and may improve patient satisfaction. The device will be more expensive than wire localization and a full cost analysis including the impact of purchase price, radiological efficiency, theatre efficiency, radionuclide injections, patient satisfaction and surgical outcomes is planned. Recent evidence suggests that radioactive seed localization is more cost effective than wire localization [9], and this saving may be comparable for Magseed surgery.

This study has provided the safety and feasibility data that have enabled this technology to be CE marked (European safety mark for medical devices), and to validate that the seed works as intended. This study has enabled the next phase of Magseed research, which is the validation of its application and outcomes in lumpectomy surgery in a larger population.

In conclusion, Magseed is a feasible means of localizing breast lesions and is safe to deploy. It is commercially available in Europe and the US and has been used in over 3000 patients. Studies are ongoing in the US and Europe to demonstrate its effectiveness in the setting of lumpectomy surgery. It will improve radiological and surgical scheduling and give surgeons intraoperative directional determination of the cancer site using the Sentimag probe. Magseed has many potential applications, including the marking of axillary lymph nodes for targeted axillary dissection and perhaps for marking of lesions in other organs.
